# Sepsis induces incomplete M2 phenotype polarization in peritoneal exudate cells in mice

**DOI:** 10.1186/s40560-015-0124-1

**Published:** 2016-01-12

**Authors:** Nobuo Watanabe, Yusuke Suzuki, Sadaki Inokuchi, Shigeaki Inoue

**Affiliations:** Department of Emergency and Critical Care Medicine, Tokai University School of Medicine, Shimokasuya 143, Isehara, Kanagawa 259-1193 Japan

**Keywords:** Macrophage, Immunosuppression, Secondary infection, Sepsis

## Abstract

**Background:**

Macrophages can differentiate into pro-inflammatory (M1) or anti-inflammatory (M2) phenotypes upon exposure to a pathogen or a cytokine microenvironment. However, M1/M2 macrophage polarization in polymicrobial sepsis has not been fully characterized.

**Methods:**

The polarity of peritoneal exudate (PE) cells from mice that had undergone cecal ligation and puncture (CLP) and the response of those cells to lipopolysaccharide (LPS) in terms of cytokine and chemokine expression were examined.

**Results:**

PE cells from CLP mice demonstrated a shift toward the M2 phenotype in terms of marker enzyme expression. In addition, the CLP-derived PE cells showed apparent unresponsiveness to LPS stimulation with regard to expression of pro-inflammatory cytokines such as TNF-α, while the expression of anti-inflammatory cytokines such as IL-10 was induced. Nevertheless, the CLP-PE cells failed to express M2 chemokines including chemokine (C-C motif) ligand 17 (CCL17), CCL22, and CCL24, all of which are important for T cell recruitment.

**Conclusions:**

The results suggested that a shift of naïve monocytes/macrophages to the M2 phenotype, along with the lack of M2 chemokine expression in septic monocytes/macrophages, might be responsible for immunosuppression after sepsis.

**Electronic supplementary material:**

The online version of this article (doi:10.1186/s40560-015-0124-1) contains supplementary material, which is available to authorized users.

## Background

Sepsis is a systemic inflammatory response to infection that can lead to organ dysfunction. It is one of the most challenging clinical problems worldwide and the leading cause of death in intensive care units [1]. After an inflammatory phase, characterized by excessive production of pro-inflammatory mediators [2], sepsis patients are thought to enter an immunosuppressive phase with impaired innate and adaptive immunity [3, 4]. In adaptive immunity, T cell exhaustion, which refers to decreased T cell numbers and function with a state of unresponsiveness to antigen-presenting cells, causes the immunosuppression associated with secondary infection after sepsis [5–7].

Besides the importance of T cells in adaptive immunity after sepsis, macrophages are crucial to innate immunity and play critical role in the response to microbial invasion in the early phase of sepsis. Microbial products or Th1 cytokines (tumor necrosis factor [TNF] and interleukin [IL]-6) polarize monocytes/macrophages toward M1 cells, which release pro-inflammatory cytokines/chemokines that provoke inflammation and contribute to the killing of bacteria, while Th2 cell cytokines (IL-4 and IL-13) polarize monocytes/macrophages to M2 macrophages, which release anti-inflammatory cytokines/chemokines and contribute to tissue repair and remodeling [8, 9]. Inducible nitric oxide synthase (iNOS) and arginase 1 (ARG1) are regarded marker enzymes for M1 and M2 macrophages, respectively [9]. Suppressor of cytokine signaling (SOCS) [10] protein levels have been recently proposed as alternative markers for macrophage polarity: a high SOCS3/SOCS1 expression ratio has been suggested as an indicator of the M1 phenotype, while high a SOCS1/SOCS3 ratio characterizes M2 macrophages [11, 12].

Previous studies have demonstrated that macrophages exposed to lipopolysaccharide (LPS) show reduced responsiveness to subsequent LPS challenge in vitro [13] and in the cecal ligation puncture (CLP) model in vivo [14]. This phenomenon, referred to as “LPS tolerance”, is associated with impaired production of pro-inflammatory cytokines [9]. However, the M1/M2 polarity and functional alterations of CLP-derived macrophages have not yet been extensively studied, especially in terms of cytokine, chemokine expression, and marker enzymes of M1/M2 macrophages. To obtain a clue to the mechanism of immune-suppression in septic patients, we analyzed peritoneal macrophages in CLP mice. The purpose of this study was to elucidate the M1/M2 polarity of macrophages in peritoneal exudate (PE) cells on the basis of messenger RNA (mRNA) and protein profiles of marker enzymes (iNOS and ARG1), suppressor of cytokine signaling (SOCS) isoforms, cytokines, and chemokines after sepsis induced by CLP.

## Methods

### Mice

Young (6- to 8-week-old) ICR mice were purchased from CLEA Japan (Tokyo, Japan). The mice were housed in groups of 5 for at least 1 week prior to use. All animal experiments were conducted in accordance with guidelines and protocols approved by the Tokai University Animal Studies Committee (reference number: 123026).

### CLP sepsis model

The CLP model developed by Chaudry et al. [15] was used to induce intra-abdominal peritonitis. In brief, the mice were anesthetized with isoflurane, and a midline abdominal incision was made. The cecum was mobilized, half-ligated below the ileocecal valve, and punctured twice using an 18-gauge needle. The abdomen was closed in two layers, and the mice were subcutaneously injected with 0.1 ml of 0.9 % saline containing 2 μg of buprenorphine for analgesia. Sham-operated control mice received the same surgical operation without cecum punctures. No fluids or antibiotics were administered to mice that underwent the operation. The mice were sacrificed by cervical dislocation at 6 or 20 h post-surgery for collection of PE cells (*n* = 4 and *n* = 5 per treatment group for sham and CLP mice, respectively).

PE cells were harvested by peritoneal lavage. Briefly, 5 ml of phosphate-buffered saline (PBS) containing 0.1 % bovine serum albumin was injected into the peritoneal cavity, the abdomen was massaged 30 times, and the PBS was recovered by using a 14-gauge catheter (Angiocath; BD Biosciences, Franklin Lanes, NJ, USA). After removing the erythrocytes by osmotic lysis, the PE cells were seeded into a 96-well plate at a density of 1 × 10^5^ cells/well and cultured in 0.2 ml of RPMI 1640 medium containing 10 % fetal calf serum, penicillin (100 U/ml) and streptomycin (100 μg/ml) in the presence or absence of LPS (1 μg/ml) for 3 h or 6 h.

### Real-time PCR

mRNA was extracted from the cultured PE cells by using Sepasol (Nacalai Tesque, Kyoto, Japan), and complementary DNA (cDNA) was synthesized using the High Capacity cDNA reverse transcription kit (Life Technologies, Carlsbad, CA, USA) according to the manufacturer’s instructions. Real-time PCR was carried out with SYBR green (Life Technologies or Toyobo Co., Osaka, Japan) using an ABI 7500 real-time thermal cycler (Applied Biosystems, Foster City, CA, USA). The 2^−ΔΔCt^ method was used to calculate the fold change in expression of each gene of interest, and *GADPH* was used as an internal control. The sequences of the PCR primers used in this study are listed in Table 1.

**Table 1:** The sequences of the PCR primers used in this study

Target gene		Sequence
TNF-α	Forward	CTGTAGCCCACGTCGTAGC
	Reverse	TTGAGATCCATGCCGTTG
IL-1β	Forward	TGTAATGAAAGACGGCACACC
	Reverse	TCTTCTTTGGGTATTGCTTGG
IL-6	Forward	GAGGATACCACTCCCAACAGACC
	Reverse	AAGTGCATCATCGTTGTTCATACA
IL-12 p35	Forward	CACCCTTGCCCTCCTAAACC
	Reverse	CACCTGGCAGGTCCAGAGA
IL-10	Forward	TGAGGCGCTGTCATCGATTTCTCCC
	Reverse	ACCTGCTCCACTGCCTTGCT
IL-1ra	Forward	TCAGATCTGCACTCAATGCC
	Reverse	CTGGTGTTTGACCTGGGAGT
iNOS	Forward	ACATCGACCCGTCCACAGTAT
	Reverse	CAGAGGGGTAGGCTTGTCTC
ARG 1	Forward	ATGGAAGAGACCTTCAGCTAC
	Reverse	GCTGTCTTCCCAAGAGTTGGG
MCP1/CCL2	Forward	ACTGAAGCCAGCTCTCTCTTCCTC
	Reverse	TTCCTTCTTGGGGTCAGCACAGAC
CCL22	Forward	GTGGCTCTCGTCCTTCTTGC
	Reverse	GGACAGTTTATGGAGTAGCTT
CCL24	Forward	TGTGACCATCCCCTCATCTTGC
	Reverse	AAACCTCGGTGCTATTGCCACG
CCL17	Forward	TAAGACCTCAGTGGAGTGTTC
	Reverse	AAATGCCTCAGCGGGAAG
GAPDH	Forward	TGCACCACCAACTGCTTAG
	Reverse	GGATGCAGGGATGATGTTC
SOCS1	Forward	GTTGTAGCAGCTTGTGTCTG
	Reverse	TGGTTTGTGCAAAGATACTG
SOCS3	Forward	TACTGAGCCGACCTCTCTC
	Reverse	AGCTGGGTCACTTTCTCATA

### Cytokine assay

For the measurement of cytokine concentrations in the culture medium, PE cell preparation and treatment were performed as described above, except that mice received a single intraperitoneal injection of 5 % (*v*/*v*) DMSO in PBS (0.02 ml/g body weight) 1 h after sham or CLP surgery, and PE cells were cultured for 24 h. The cytokine concentrations in 24-h cell culture medium were measured using the Cytometric Bead Array (CBA; BD Biosciences) and fluorescence-activated cell sorting (FACS) according to the manufacturer’s instructions.

### Statistical analysis

The data are expressed as the mean ± SEM and were analyzed using IBM SPSS Statistics version 20 (SPSS, Chicago, IL, USA). Multiple experimental groups were compared using one-way ANOVA and Tukey’s post hoc test. Two-way ANOVA was performed to determine the main effects of LPS and CLP, as well as the interaction between these two factors. A *P* value < 0.05 was defined as statistically significant.

## Results

### Sepsis increases the number of PE cells without affecting the monocyte/macrophage ratio

The number of PE cells recovered from mice with CLP at 20 h after surgery was doubled compared to that from sham mice (7.4 × 10^6^ in CLP vs. 3.7 × 10^6^ in sham). FACS analysis revealed that the number of lymphocytes was significantly decreased in the CLP-PE cell population compared to the control, which was consistent with previously reported results [6]. However, the monocyte/macrophage ratio in the PE cell population was 30–40 % in both the sham and CLP groups (data not shown).

### CLP polarizes PE cells initially to the M1, and subsequently to the M2 phenotype

**Fig. 1:**
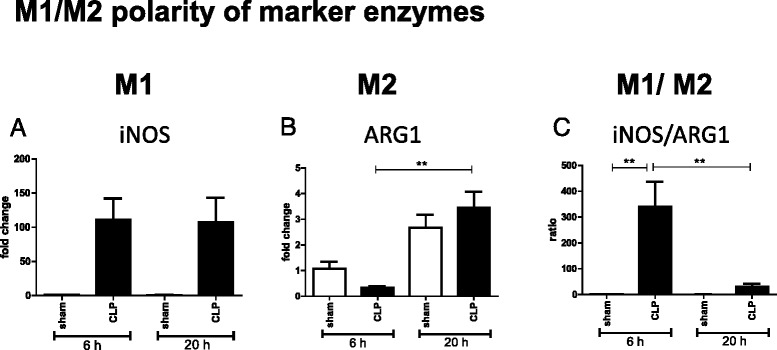
CLP polarized PE cells initially to the M1, and subsequently to the M2 phenotype. PE cells were harvested from mice at 6 or 20 h after sham or CLP operation (*n* = 4 or 5 for each group). The mRNA expression levels of the marker enzymes iNOS (**a**) and ARG1 (**b**) were analyzed by real-time PCR. The fold changes are expressed relative to sham-PE cells harvested at 6 h post-surgery. In **c**, the ratio of iNOS/ARG1 is shown. Data are presented as the mean + SEM. **P < 0.01, *P < 0.05, CLP vs. sham animals

The levels of iNOS mRNA were significantly increased in CLP-PE cells as compared with sham-PE cells at both 6 and 20 h after sham or CLP (*P* < 0.01; Fig. 1a). There was no difference in the iNOS mRNA levels at 6 and 24 h post-CLP. In contrast, the ARG1 level at 6 h post-surgery was significantly lower in CLP-PE than in sham-PE cells (Fig. 1b). Further, the ARG1 level at 20 h post-surgery was significantly higher than that at 6 h post-surgery (*P* < 0.01; Fig. 1b). As a result, the ratio of iNOS to ARG1 was significantly higher in CLP-PE cells at 6 h than at 20 h post-surgery, indicating that CLP-PE cells were polarized to the M1 phenotype at 6 h, and then to the M2 phenotype at 20 h after CLP (Fig. 1c).

CLP increased both the levels of SOCS3 and SOCS1 (Additional file 1: Figure S1 A and B), irrespective of the post-surgery period. However, no significant differences were observed for the SOCS3/SOCS1 ratio between sham and CLP, or between 6 and 20 h post-surgery (Additional file 1: Figure S1 C). Thus, CLP initially skewed PE cells to M1 (at 6 h), and later (at 20 h) to the M2 phenotype according to the iNOS/ARG ratio, while the SOCS3/SOCS1 ratio did not provide any information regarding polarity within this post-surgery period.

### Cytokine expression shifts toward the M2 phenotype with LPS stimulation of CLP-derived PE cells

**Fig. 2:**
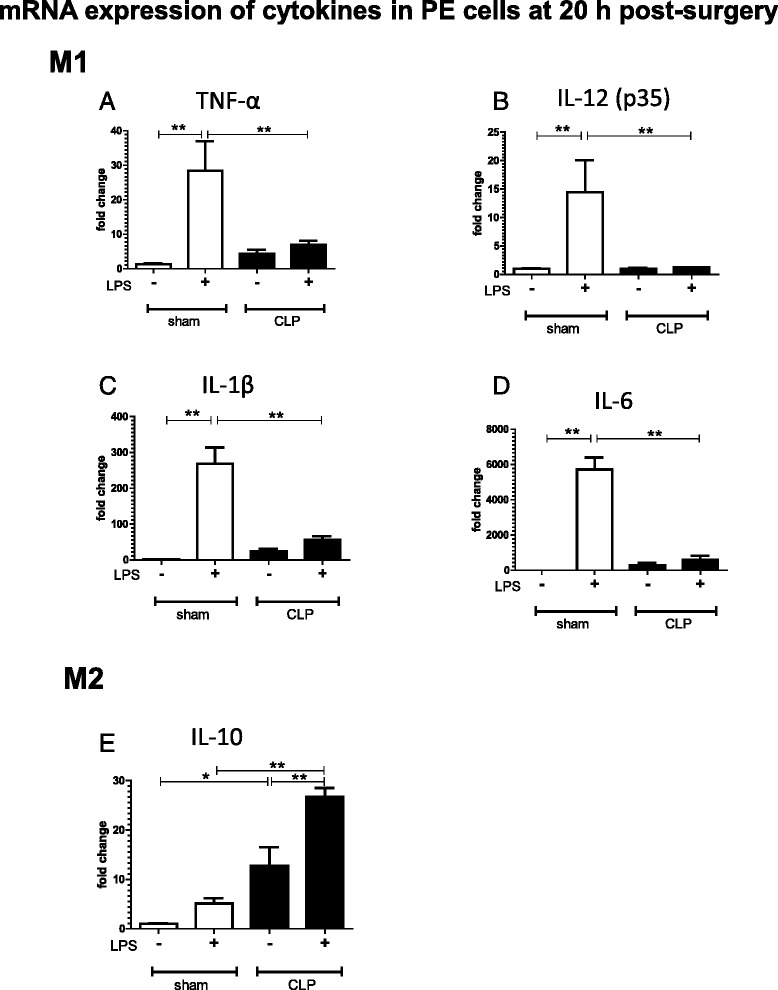
M2 polarization of cytokine expression of CLP-derived PE cells after LPS stimulation. PE cells harvested from mice at 20 h after sham or CLP operation were cultured in the presence or absence of LPS (1 μg/ml) for 6 h (*n* = 4 or 5 for each group). Real-time PCR was used to analyze the expression of the M1 cytokines TNF-α (**a**), IL-12 (**b**), IL-1β (**c**), and IL-6 (**d**), and the M2 cytokine IL-10 (**e**). The fold changes are expressed with respect to sham-PE cells without LPS stimulation. Data are presented as the mean + SEM. **P < 0.01, *P < 0.05, CLP vs. sham animals

LPS-induced robust expression of the M1 cytokine TNF-α in sham-PE, but not in CLP-PE cells (Fig. 2a). In both 6-h- and 20-h-post-surgery CLP-PE cells, TNF-α expression reached a peak at 3 h after LPS stimulation, and then declined over the next 3 h (Additional file 2: Figure S2). Similar to TNF-α, LPS-induced potent expression of the pro-inflammatory cytokines IL-1β, IL-6, and IL-12 (p35 subunit) in sham-PE, but not in CLP-PE cells (Fig. 2b–d).

CLP-PE cells expressed higher levels of the M2 cytokine IL-10 than sham-PE cells, either 6 or 20 h post-surgery (Additional file 2: Figure S2 C and D). In contrast to M1 cytokines, CLP and LPS treatment induced a significant increase in IL-10 expression in PE cells when compared to sham-PE cells (*P* < 0.05; Fig. 2e). Taken together, these results indicate that CLP-derived PE cells polarize to M2, with decreased M1 and increased M2 cytokine expression, in response to LPS treatment.

### Chemokine expression shifts toward M1 phenotype with LPS stimulation of CLP-derived PE cells

**Fig. 3:**
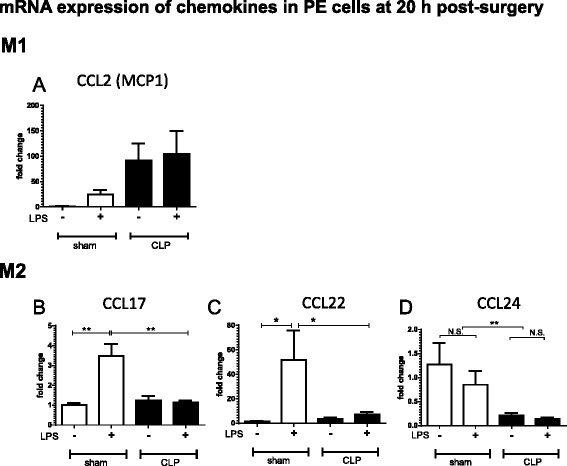
M1 polarization of chemokine expression of CLP-derived PE cells after LPS stimulation. PE cells harvested from mice at 20 h after sham or CLP operation were cultured in the presence or absence of LPS (1 μg/ml) for 6 h (*n* = 4 or 5 for each group). Real-time PCR was used to analyze the expression of the M1 chemokine CCL2 (**a**) and the M2 chemokines CCL17 (**b**), CCL22 (**c**), and CCL24 (**d**). The fold changes are expressed with respect to sham-PE cells without LPS stimulation. Data are presented as the mean + SEM. **P < 0.01, *P < 0.05, CLP vs. sham animals

Chemokine (C-C motif) ligand 2 (CCL2/MCP1) is a typical M1 macrophage chemokine [16, 17]. CLP-PE cells derived from mice at 20 h post-surgery showed a trend of elevated CCL2 without LPS treatment (Fig. 3a). Similar to cytokine expression, LPS treatment induced CCL2 expression in sham-PE cells. However, in CLP-PE cells, no further increase in CCL2 expression was observed after LPS stimulation.

CCL17, CCL22, and CCL24 have been identified as M2 macrophage chemokines [9, 17, 18]. LPS-induced expression kinetics of CCL22 was measured in PE cells from 6-h- and 20-h-post-surgery mice. LPS-induced CCL22 expression was severely suppressed in CLP-PE cells, especially in those derived from 20-h-post-CLP mice (Additional file 2: Figure S2 E and F). In a separate experiment with 6-h LPS treatment of PE cells from mice at 20 h post-surgery, LPS treatment also caused a marked increase in both CCL17 and CCL22 in sham-PE, but not in CLP-PE cells (Fig. 3b, c). CLP-PE cells showed lower CCL24 expression than sham-PE cells, regardless of LPS stimulation (Fig. 3d). Thus, while M1 chemokine expression was higher in CLP-PE than in sham-PE cells regardless of LPS treatment, M2 chemokine expression upon LPS treatment was suppressed. These results indicated that the CLP-PE cells had shifted to the M1 phenotype with regard to their chemokine expression profile.

### Supernatant cytokine shifts toward M2 phenotype with LPS stimulation of CLP-derived PE cells

**Fig. 4:**
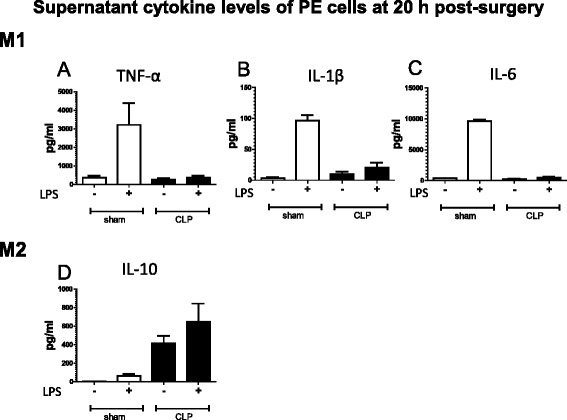
M2 polarization of supernatant cytokine incubated CLP-derived PE cells with LPS stimulation. PE cells harvested from mice at 20 h after sham or CLP operation were cultured in the presence or absence of LPS (1 μg/ml) for 24 h (*n* = 2 for sham and *n* = 3 for CLP). The levels of cytokines TNF-α (**a**), IL-1β (**b**), IL-6 (**c**), and IL-10 (**d**) in the cell culture medium were measured by CBA assay kit and FACS. Data are presented as the mean + SEM

LPS stimulation increased the levels of TNF-α, IL-1β, and IL-6 in sham-PE, but not in CLP-PE cells (Fig. 4a–c). In contrast, significant amounts of the M2 cytokine IL-10 were detected in the culture medium of CLP-PE cells, regardless of LPS stimulation. Thus, the supernatant cytokine levels with LPS treatment of CLP-PE cells indicated a shift to the M2 phenotype, which was consistent with the mRNA analysis results.

## Discussion

The current study revealed that CLP-derived PE cells polarized to M2 phenotype in terms of marker enzyme expression and showed an M2 cytokine expression profile upon LPS stimulation. However, the chemokine expression profile suggested a shift to the M1 phenotype, which is related to T cell recruitment. The results may explain the underlying mechanism of immunosuppression after sepsis.

Recently, persistent inflammation and immunosuppression have been observed in a genome-wide expression analysis of severely injured patients [5, 19]. Our previous studies demonstrated that T cell exhaustion is one of the causes of immunosuppression in elderly sepsis patients suffering from secondary infections [6, 7]. To gain more insight into immunosuppression in sepsis patients, this study focused on the differentiation of PE macrophages into M1/M2 phenotypes after CLP and their response to LPS. According to the iNOS/ARG1 ratio, CLP initially polarized PE cells to the M1 phenotype (at 6 h after CLP), and subsequently to the M2 phenotype (at 20 h after CLP), which is consistent with a conceptual immunological framework indicating that immunosuppression is more severe in the late than in the early phase after sepsis [3]. However, both SOCS3 and SOCS1 were equally induced by CLP, and no difference was observed in the SOCS3/SOCS1 ratio. In a study on peritoneal macrophages from CLP mice, SOCS3 was upregulated whereas SOCS1 remained unchanged [16, 17]. This discrepancy may be attributed to the different mouse strains used (ICR in our study vs. 3H/HeN in the previous studies) and/or assayed parameters (mRNA level in our study vs. protein level in the previous studies).

Similar to previously reported cases of LPS tolerance [13, 20], our study demonstrated that CLP suppressed M1 cytokine and increased M2 cytokine expression in PE cells upon subsequent challenge with LPS in vitro. Further, we confirmed an M2-type-dominated cytokine protein expression profile: the concentrations of TNF-α, IL-1β, and IL-6 in culture media containing CLP-PE cells were significantly lower than that in media containing sham-PE cells after treatment with LPS for 24 h, whereas the concentration of IL-10 was higher in CLP-PE cell-derived culture media. The cytokine protein expression profiles were consistent with those reported by Ayala et al. [14], who also demonstrated an M2 shift in cytokine expression in LPS-treated peritoneal macrophages from CLP mice. Taken together, the mRNA and protein profiles clearly suggested that CLP-PE cells are polarized to the M2 phenotype in terms of LPS-induced cytokine expression.

However, the LPS-induced chemokine expression profile of the CLP-PE cells unexpectedly indicated an M1 phenotype. Previous studies have reported contradictory chemokine expression profiles in LPS-tolerant macrophages. Porta et al. [20] reported that LPS challenge in LPS-tolerant macrophages induced both M1 (CCL2) and M2 chemokines (CCL17, CCL22) in vitro, whereas Rajaiah et al. [13] showed downregulation of these markers in similar experimental conditions. However, these studies are in vitro culture experiences primed and challenged by LPS [13, 20]. Our study ex vivo demonstrated a complex mechanism for macrophage sensitization after polymicrobial sepsis. Additionally, we showed that CCL24 expression was downregulated by CLP.

Because M2 chemokines play an important role in the recruitment of T cells [9, 18, 21–23], the inability of CLP-PE cells to express M2 chemokines in response to microbial LPS might impair the elicitation of a subsequent adaptive immune response against pathogenic bacteria after sepsis. Therefore, a shift toward the anti-inflammatory M2 phenotype in terms of cytokine expression but the inability to express M2 chemokines in macrophages as revealed in this study might underlie the immunosuppression associated with secondary infection after sepsis.

Our study had several limitations. First, we examined the response in PE cells, not in isolated macrophages. Second, only mRNA expression was evaluated, except for several cytokines for which the protein concentrations in cell culture media were assessed. Several chemokines related to M1 and M2 polarization were technically not measurable in this study, while measurement of cytokines and chemokines in peritoneal lavage fluids would be needed to confirm our results. Third, we did not elucidate the molecular mechanism underlying the phenotypic shift of PE cells induced by CLP. Recent studies have suggested the importance of the PI3 kinase pathway in the phenotypic shift in general [24], and we are currently investigating the role of the PI3 pathway in the CLP-induced phenotypic shift of PE cells. Additional in-depth studies are also needed to assess the impact of the phenotypic shift to M2-type macrophages in overall immunosuppression, which leads to secondary infection after sepsis. While this study showed that immunosuppression after sepsis occurs in monocytes/macrophages as well as in T cells, further study is needed to reveal dysfunction of monocytes/macrophages after sepsis.

## Conclusions

Sepsis induces an incomplete polarization to the M2 phenotype in PE cells in a mouse model. The M2 shift in cytokines and marker enzymes along with the lack of M2 chemokine expression in septic monocytes/macrophages might be related to immunosuppression with increased secondary infection after sepsis.

## Additional files

Additional file 1: Figure S1. SOCS1 and SOCS3 expression in PE cells after CLP. PE cells were harvested from mice at 6 h or 20 h after sham or CLP operation (*n* = 4 or 5 for each group). The mRNA expression levels of marker enzymes SOCS3 (A) and SOCS1 (B) were analyzed by real-time PCR. The fold changes are expressed relative to sham-PE cells harvested at 6 h post-surgery. In (C), the ratio of SOCS3/SOCS1 is shown. Data are presented as the mean ± SEM. ***P* <0.01, **P* <0.05, CLP vs. sham animals. (PDF 389 kb) Additional file 2: Figure S2. Time course of LPS-induced expression of M1 and M2 cytokines/chemokines in PE cells. PE cells harvested from mice at 6 h (A, C, and E) and 20 h (B, D, and F) after sham or CLP operation were cultured in the presence or absence of LPS (1 μg/ml) for 0 h, 3 h, and 6 h (*n* = 4 or 5 for each group). Real-time PCR was used to analyze the expression of TNF-α (A and B), IL-10 (C and D), and CCL22 (E and F). The fold changes are expressed relative to the expression levels at 0 h of sham-PE cells obtained at 6 h post-surgery. (PDF 424 kb)

## Abbreviations

ARG: arginase
CCL: chemokine (C-C motif) ligand
CLP: cecal ligation and puncture
IL: interleukin
iNOS: inducible nitric oxide synthase
LPS: lipopolysaccharide
MCP: monocyte chemoattractant protein
PE: peritoneal exudate
SOCS: suppressor of cytokine signaling
