# Supplementary figures and images for: Sepsis induces incomplete M2 phenotype polarization in peritoneal exudate cells in mice
Source: J Intensive Care. 2016 Jan 12;4:6. doi: 10.1186/s40560-015-0124-1 (PMC4709882; doi:10.1186/s40560-015-0124-1)

# SOCS1 and SOCS3 expression in PE cells after CLP

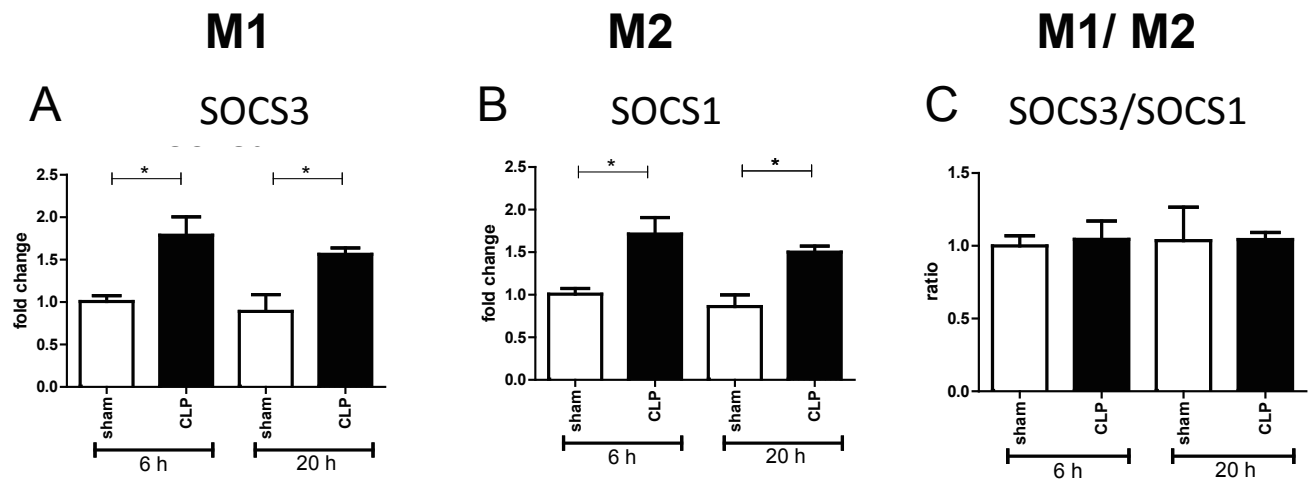

Supplement: Additional file 1: Figure S1. — SOCS1 and SOCS3 expression in PE cells after CLP. PE cells were harvested from mice at 6 h or 20 h after sham or CLP operation (n = 4 or 5 for each group). The mRNA expression levels of marker enzymes SOCS3 (A) and SOCS1 (B) were analyzed by real-time PCR. The fold changes are expressed relative to sham-PE cells harvested at 6 h post-surgery. In (C), the ratio of SOCS3/SOCS1 is shown. Data are presented as the mean ± SEM. **P <0.01, *P <0.05, CLP vs. sham animals. (PDF 389 kb) [file 40560_2015_124_MOESM1_ESM.pdf]
