# Supplementary material for: Sepsis induces incomplete M2 phenotype polarization in peritoneal exudate cells in mice
Source: J Intensive Care. 2016 Jan 12;4:6. doi: 10.1186/s40560-015-0124-1 (PMC4709882; doi:10.1186/s40560-015-0124-1)

Supplemental Figure 2

Time course of mRNA expression of cytokines in PE cells

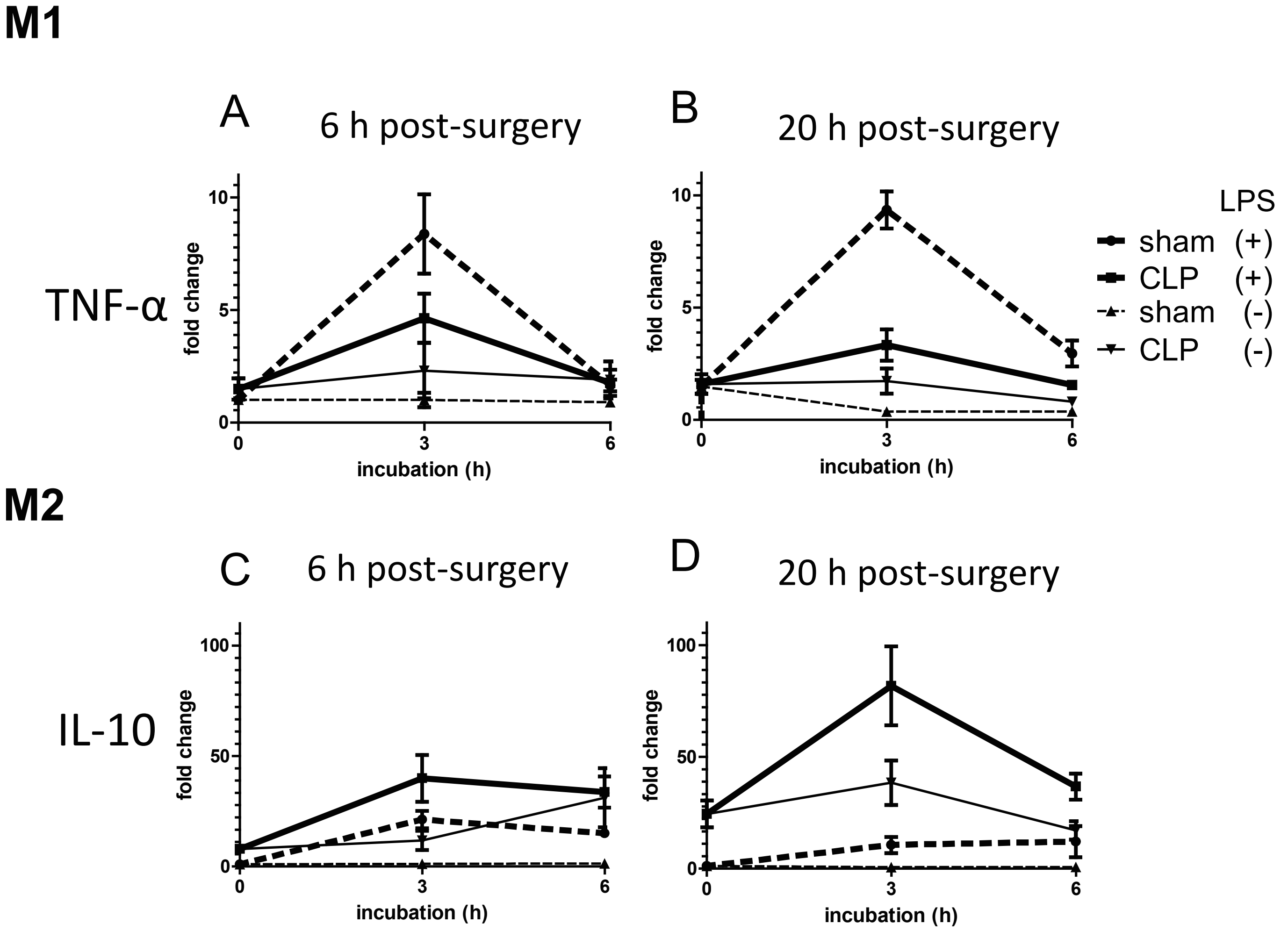

Time course of mRNA expression of chemokine in PE cells

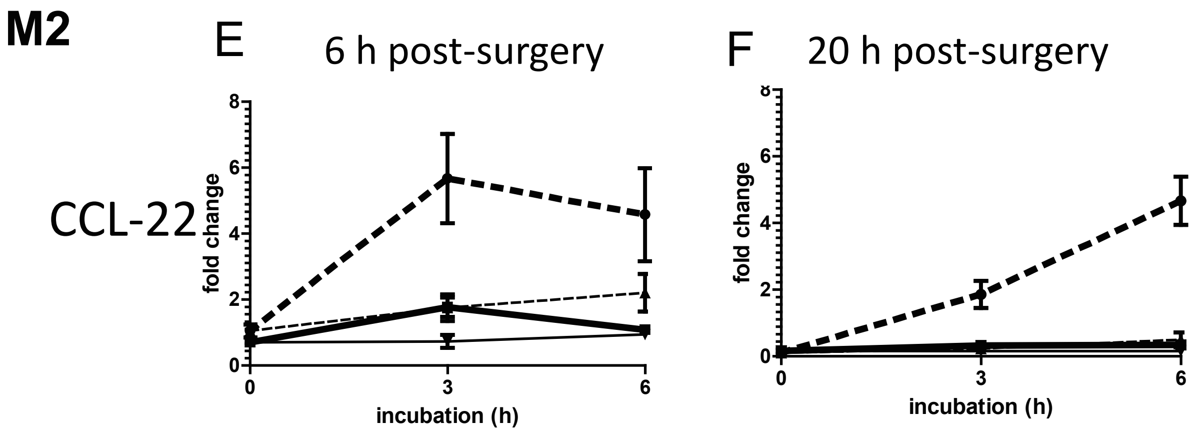

Supplement: Additional file 2: Figure S2. — Time course of LPS-induced expression of M1 and M2 cytokines/chemokines in PE cells. PE cells harvested from mice at 6 h (A, C, and E) and 20 h (B, D, and F) after sham or CLP operation were cultured in the presence or absence of LPS (1 μg/ml) for 0 h, 3 h, and 6 h (n = 4 or 5 for each group). Real-time PCR was used to analyze the expression of TNF-α (A and B), IL-10 (C and D), and CCL22 (E and F). The fold changes are expressed relative to the expression levels at 0 h of sham-PE cells obtained at 6 h post-surgery. (PDF 424 kb) [file 40560_2015_124_MOESM2_ESM.pdf]
